# The lncRNA MALAT1 is a novel biomarker for gastric cancer metastasis

**DOI:** 10.18632/oncotarget.10941

**Published:** 2016-07-29

**Authors:** Hongwei Xia, Qingjuan Chen, Ying Chen, Xiaojun Ge, Weibing Leng, Qiulin Tang, Ming Ren, Liang Chen, Dandan Yuan, Yucheng Zhang, Ming Liu, Qiyong Gong, Feng Bi

**Affiliations:** ^1^ Laboratory of Signal Transduction & Molecular Targeted Therapy, State Key Laboratory of Biotherapy and Cancer Center, West China Hospital, Sichuan University, and Collaborative Innovation Center for Biotherapy, Chengdu, Sichuan Province, China; ^2^ Department of Medical Oncology, West China Hospital, Sichuan University, Chengdu, Sichuan Province, China; ^3^ Department of Medical Oncology, Xian Yang Central Hospital, Xian Yang City, Shanxi Province, China; ^4^ Department of Radiology, West China Hospital of Sichuan University, Chengdu, Sichuan Province, China

**Keywords:** long non-coding RNA, MALAT1, gastric cancer, metastasis, miR-122

## Abstract

The metastasis-associated lung adenocarcinoma transcript 1 (MALAT1) is frequently over-expressed and serves as a prognostic marker in human cancers. However, little is known about the role of MALAT1 in gastric cancer. Here, we reported that the tissue and plasma MALAT1 levels were significantly higher in gastric cancer patients with distant metastasis (P<0.01) than patients without distant metastasis and the healthy controls. In addition, high levels of plasma MALAT1 independently correlated to a poor prognosis for gastric cancer patients (hazard ratio, 0.242; 95% CI, 0.154-0.836; P=0.036; Cox regression analysis). Functional studies revealed that knockdown of MALAT1 could inhibit cell proliferation, cell cycle progression, migration and invasion, and promote apoptosis in gastric cancer cells. Furthermore, the miR-122-IGF-1R signaling correlated with the dysregulated MALAT1 expression in gastric cancer. These data suggest that MALAT1 could function as an oncogene in gastric cancer, and high MALAT1 level could serve as a potential biomarker for the distant metastasis of gastric cancer.

## INTRODUCTION

Gastric cancer (GC) is the fourth common cancer and the second leading cancer-related cause of death worldwide [1, 2]. Although the early detection rate of GC have increased, there are still many patients suffering from distant metastasis, with a median survival of only 3-5 months [1-4]. Thus, it is important to identify oncogenes that promote gastric cancer metastasis. These oncogenes may serve as not only biomarkers for gastric cancer progression, but also molecular targets for gastric cancer therapy.

The long non-coding RNAs (lncRNAs) are generally defined as transcripts composed of more than 200 nucleotides in length, and associated with many important cellular processes and pathogenesis. It has been reported that lncRNAs are differentially expressed in many types of tumors [5, 6]. Several studies demonstrated that lncRNAs were remarkably stable in blood samples, thus, making the quantitative detection of lncRNAs in blood feasible [7, 8]. The circulating lncRNAs have been extensively studied as biomarkers for the diagnosis and prognosis of many cancer types, including lung cancer, liver cancer, renal cell carcinoma, breast cancer, cervical cancer, uterine endometrial, stromal sarcoma and colorectal carcinoma [9-19]. However, the specific lncRNAs that are associated with GC remain poorly understood [9, 20, 21].

Metastasis-associated lung adenocarcinoma transcript 1 (MALAT1), also known as nuclear-enriched abundant transcript2 (NEAT2), is one of the first cancer-associated lncRNAs. MALAT1 is highly conserved across species and is widely expressed in human tissues [22]. Since it was discovered nearly a decade ago, an accumulated large amount of data have linked MALAT1 to many cancer types [10, 12-16, 19, 23]. Much evidence, including our previous work, have indicated that circulating microRNAs were promising biomarkers for the diagnosis and prognosis of gastric cancer and other diseases [24]. However, MALAT1 has been poorly studied for this purpose [9, 20, 21, 25]. Here, we showed that tissue and plasma MALAT1 levels were dramatically higher in GC patients with distant metastasis than patients without metastasis. Knockdown MALAT1 significantly inhibited the malignant behaviors of gastric cancer cells. The miR-122-IGF-1R signaling correlated with the deregulated MALAT1 expression in gastric cancer cells.

## RESULTS

### MALAT1 levels are up-regulated in GC/DM plasma and tissues

**Figure 1:**
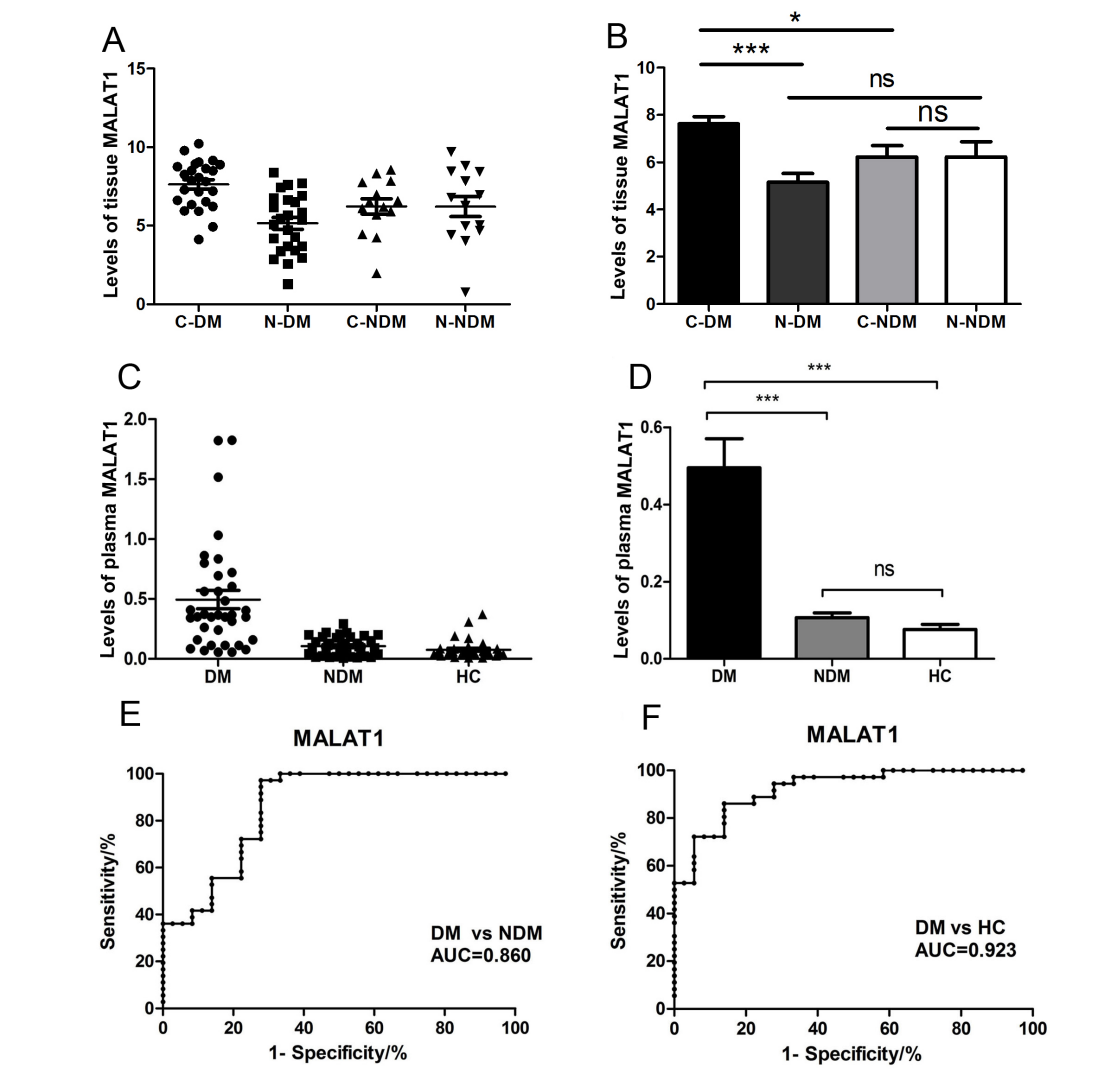
Relative tissue and plasma MALAT1 expression levels and its clinical significance in gastric cancer patients and healthy controls (**A**), (**B**) The expression of MALAT1 in tissue samples was significantly higher in the GC/DM group when compared with that of GC/NDM tissues and adjacent normal tissues. Tissue MALAT1 was detected in 14 pairs of GC/NDM patients and 25 pairs of GC/NDM patients by QRT-PCR. C-DM represents the original cancer tissues from GC/DM patients and the N-DM represents the adjacent normal tissues of the GC/DM patients. C-NDM represents the original cancer tissues from GC/NDM patients and the N-NDM represents the adjacent normal tissues of the GC/NDM patients. Data are presented as fold change. (**C**), (**D**) The expression of plasma MALAT1 was significantly higher in the GC/DM group when compared with the expression in the GC/NDM and HC groups. Plasma MALAT1 was detected in 72 pairs of patients with gastric cancer and 36 healthy controls by Q-PCR. Data are presented as fold change, and MALAT1 expression was significantly higher in patients at advanced pathological stages. (**E**), (**F**) Diagnostic efficiency of MALAT1 in the three groups. The AUC of the ROC curve for detecting DM from NDM was 0.860 (P<0.0001). The AUC of the ROC curve for detecting DM from HC was 0.923 (P<0.0001). GC, gastric cancer; GC/DM, GC with distant metastasis; GC/NDM, GC with no distant metastasis; HC, healthy controls; AUC, area under the curve; ROC, receiver operating characteristic. Values were normalized to those for β-actin. Data represent the mean ± S.D. *P< 0.05, ***P < 0.001.

Here, we firstly collected 39 pairs of gastric cancer tissues and adjacent normal tissues, including 14 pairs gastric cancer patients without distant metastasis(GC/NDM) and 25 pairs gastric cancer patients with distant metastasis(GC/DM), and extracted the RNAs from these tissues. Q-PCR was performed to detect the levels of MALAT1 in the tissues, and the results indicated that the MALAT1 levels was significantly higher in GC/DM cancer tissues (C-DM) than that of the adjacent normal tissues (N-DM) (*P*< 0.0001) and the GC/NDM cancer tissues (C-NDM) (*P*=0.0134), while there is nearly no difference between the GC/NDM tissues and the adjacent normal tissues (Figure 1A, 1B, Supplementary Figure S1). Next, we systematically examined the MALAT1 levels in the plasma of 36 healthy controls (HC) and 72 gastric cancer patients, including 36 GC/NDM and 36 GC/DM. Interestingly, the result is consistent with the data in tissues. The levels of plasma MALAT1 was significantly higher (*P*<0.01) in the GC/DM when compared with that of the GC/NDM and HC groups, and there was nearly no difference in the levels of plasma MALAT1 between the GC/NDM and HC groups (*P*>0.05) (Figure 1C and 1D, Supplementary Figure S2). Using the AUC of the ROC curve to estimate the diagnostic value of plasma MALAT1 in discerning distant metastasis in GCs, we found that the plasma levels of MALAT1 could effectively distinguish patients with GC/DM from patients with GC/NDM and the HCs (Figure 1C and 1D). Univariate Cox analysis revealed that TNM stage (IV) and differentiation (poor) were significantly associated with reduced patient survival, while high levels of plasma MALAT1 correlated to poor prognosis of patients with GC. Multivariate Cox analysis indicated that high plasma MALAT1 independently correlated to a poor prognosis of patients with GC (hazard ratio, 2.169; 95% CI, 0.265 to 4.324; *P*=0.031) (Table1).

**Table 1:** Univariate and multivariate analysis of plasma MALAT1 and clinicopathologic factors associated with survival in gastric cancer.

	Univariate analysis			Multivariate analysis		
	Hazard Ratio	95% CI	*p*-value	Hazard Ratio	95% CI	*p*-value
Differentiation (Poor)	4.239	1.065 to 8.716	0.027	3.349	0.793 to 5.641	0.043
TNM stage(IV)	3.484	1.209 to 5.387	0.043	2.874	1.136 to 6.145	0.037
Metastasis location(lung)	2.459	0.756 to 5.321	0.082	(−)	(−)	(−)
Plasma MALAT1 level (high)	3.263	0.219 to 5.096	0.033	2.169	0.265 to 4.324	0.031

### MALAT1 is highly expressed in GC cell lines

**Figure 2:**
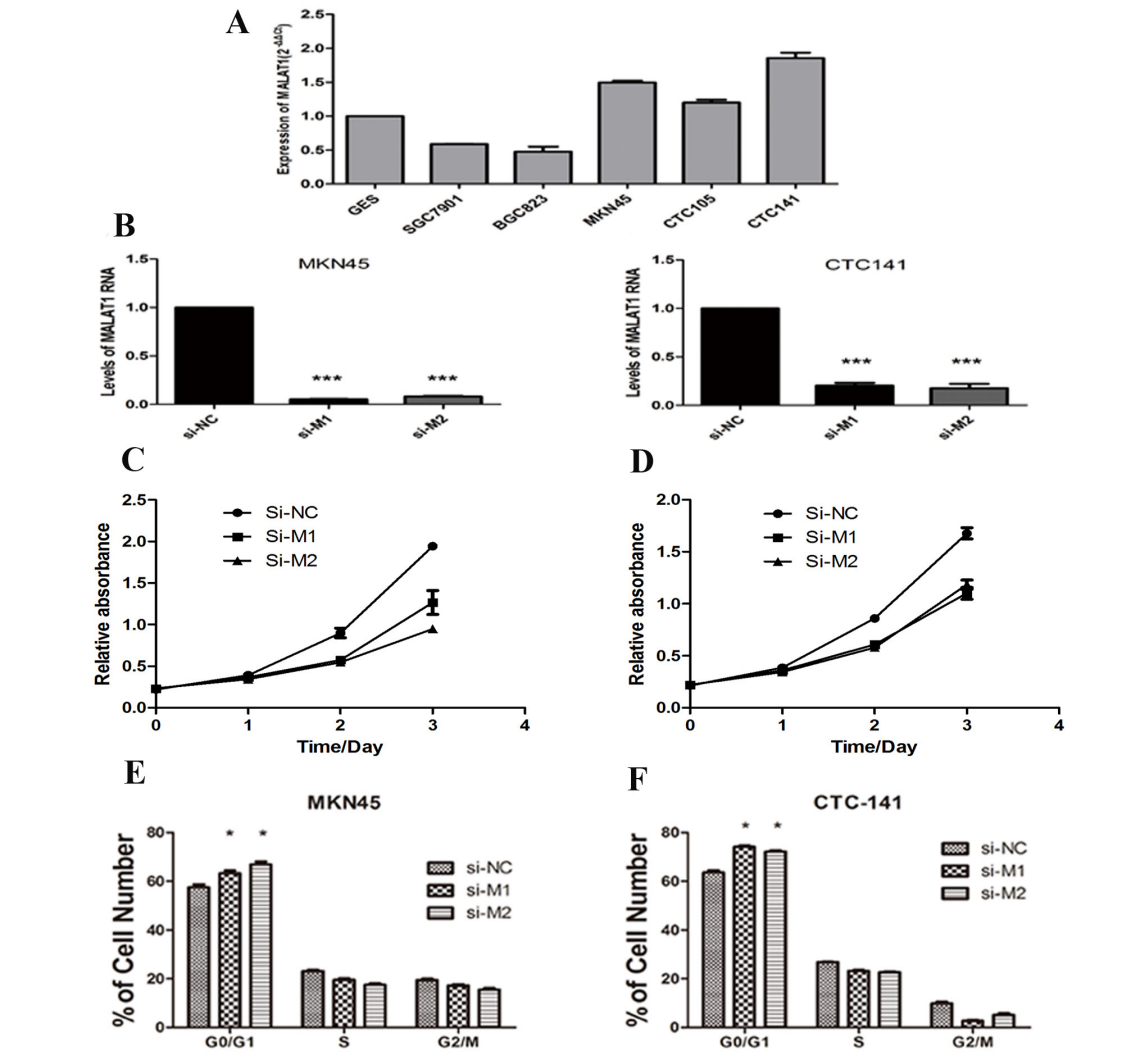
MALAT1 expression levels in gastric cancer cell lines and the effect of MALAT1 on cell proliferation (**A**) qRT-PCR analysis of MALAT1 expression levels in gastric cancer cell lines (SGC7901, BGC823, MKN45, CTC105 and CTC141) compared with the normal bronchial epithelial cell line (GES-1). (**B**) qRT-PCR analysis of MALAT1 expression following treatment of MKN45 and CTC141 cells with two individual siRNAs targeting MALAT1. (**C**), (**D**) MKN45 and CTC141 cells were transfected with si-MALAT1 or si-NC, CCK8 assays were performed to determine the proliferation of MKN45 and CTC141 cells. (**E**), (**F**) Flow cytometry was used to examine the cell cycle progression of MKN45 and CTC141 cells transfected with si-MALAT1 or si-NC. Data represent the mean ± S.D. from three independent experiments. *P< 0.05, ***P < 0.001.

Next, we performed Q-PCR to examine the expression of MALAT1 in gastric cancer cell lines. The results showed that MALAT1 was expressed in all the 6 cell lines, and significantly over-expressed in poorly differentiated gastric cancer cell lines, including MKN45, CTC141 and CTC105 (Figure 2A). To determine the biological role of MALAT1 in gastric cancers, we modulated its expression through RNA interference in MKN45 and CTC141 cells, the MALAT1 high-expressed gastric cancer cell lines. Two individual MALAT1 siRNAs were transfected into the two gastric cancer cell lines respectively. MALAT1 expression was significantly down-regulated (*P*<0.005) in the si-MALAT1 transfected cells, compared with that of the control cells (Figure 2B).

### Depletion of MALAT1 inhibits the proliferation, cell cycle progression and promotes apoptosis in gastric cancer cells

**Figure 3:**
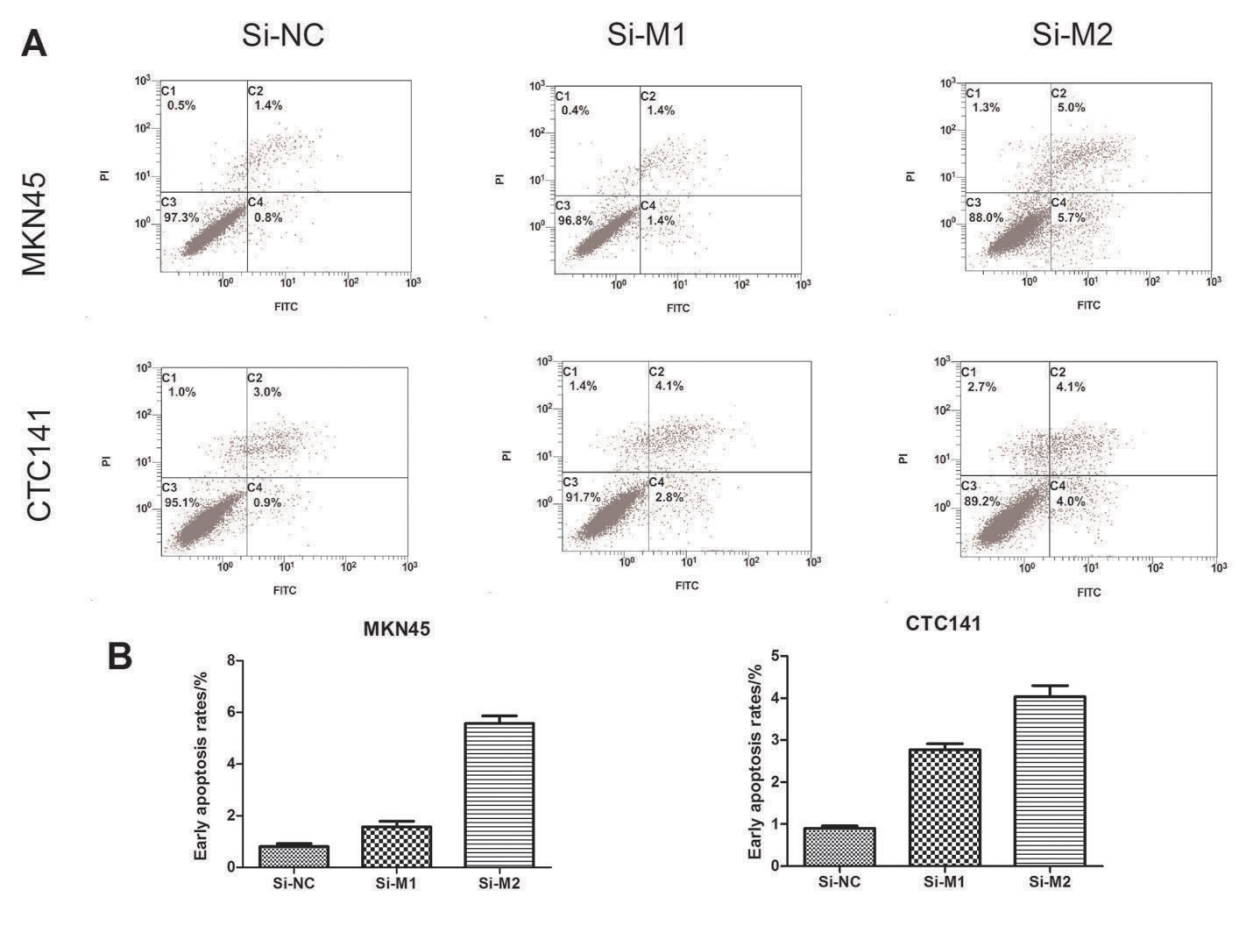
Effect of MALAT1 on cell apoptosis (**A**), (**C**) MKN45 cells were transfected with si-MALAT1 or si-NC. Early apoptotic rates were detected by flow cytometry. (**B**),(**D**) Early apoptotic rates of CTC141 transfected with si-MALAT1 or si-NC. Data represent the mean ± S.D. from three independent experiments *P< 0.05.

To study the biological function of MALAT1 in gastric cancers, we investigated the effect of MALAT1 knockdown on cell proliferation and cell cycle progression in gastric cancer cells. CCK8 assays indicated MALAT1 knockdown inhibited cell growth (Figure 2C, 2D), further flow cytometry analysis revealed that MALAT1 knockdown promoted G0/G1 phase arrest in both MKN45 and CTC141 cells (Figure 2E, 2F). Moreover, down-regulation of MALAT1 promoted apoptosis in the two gastric cancer cell lines. (Figure 3A, 3B)

### Knockdown MALAT1 suppresses the migration and invasion in gastric cancer cells

**Figure 4:**
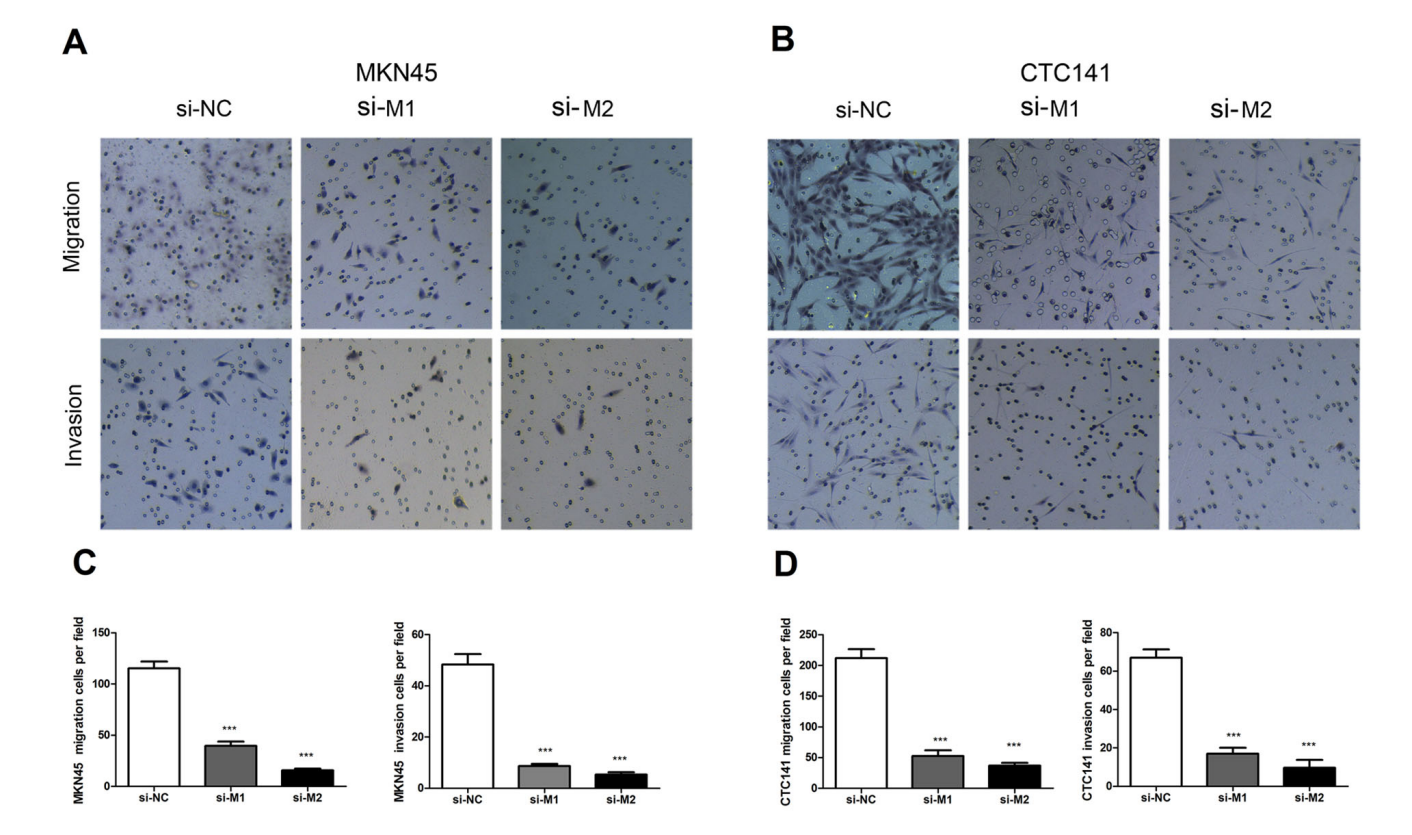
Effect of MALAT1 on cell migration and invasion (**A**, **C**) MKN45 cells were transfected with MALAT1 siRNA or si-NC. Transwell assays were performed to investigate the migratory and invasive ability. (**B**), (**D**) The migratory and invasive ability of CTC141 were detected by transwell assays. Data represent the mean ± S.D. from three independent experiments. ** P <0.01.

To investigate whether MALAT1 facilitates gastric cancer cell migration and invasion, we evaluated cancer cell migration through transwell and invasion through matrigel. As it shown in Figure 4, the migration and invasion ability was significantly reduced by approximately 65% following inhibition of MALAT1 in MKN45 (Figure 4A, 4C) and CTC141 (Figure 4B, 4D) cells (*P*<0.005).

### The miR-122-IGF-1R signaling correlated with the dysregulation of MALAT1 in gastric cancer cell

**Figure 5:**
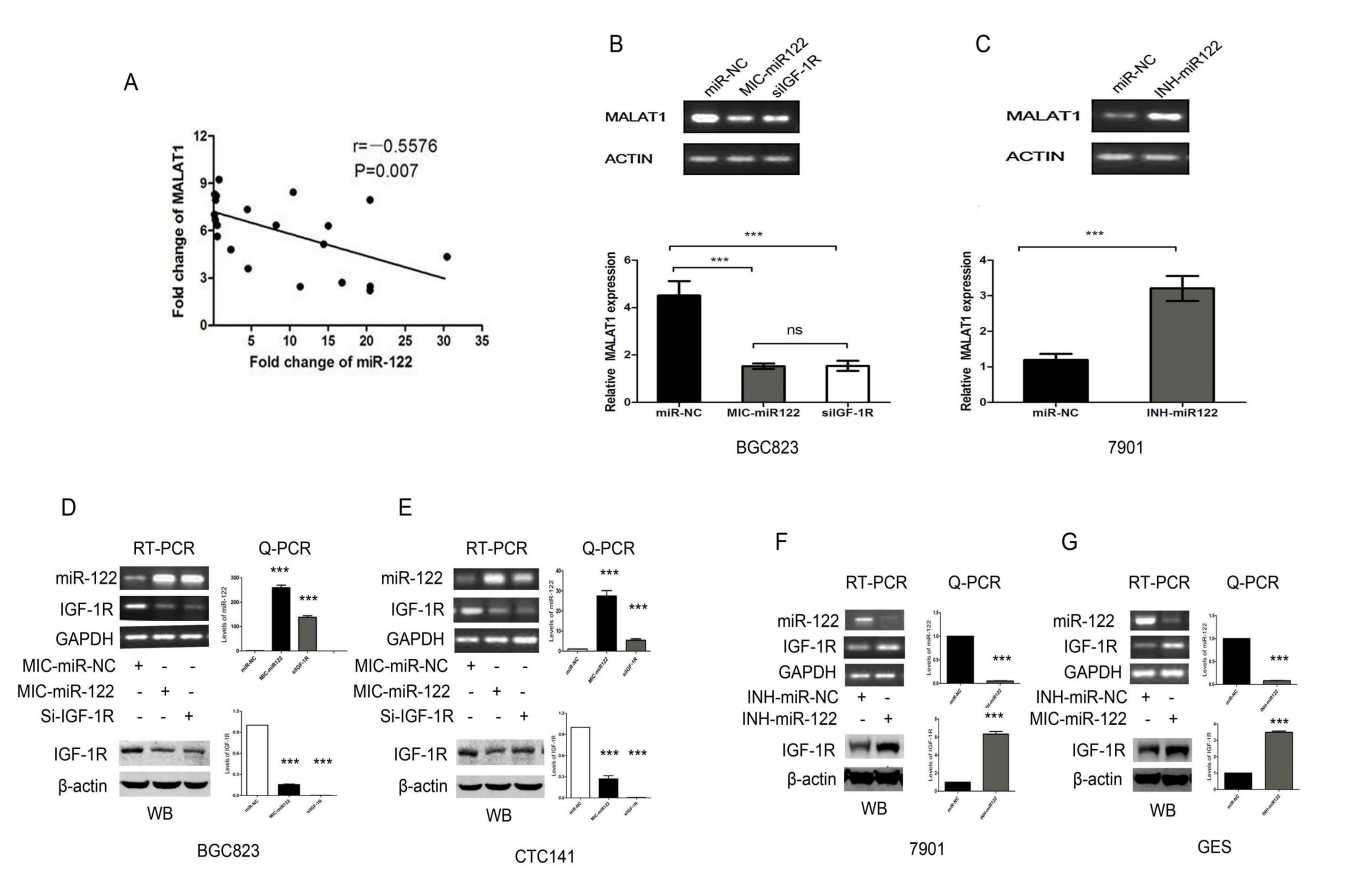
The miR-122-IGF-1R signaling might participate in the dysregulated MALAT1 expression in gastric cancer (**A**) Plasma levels of MALAT1 and miR-122 is negatively correlated in the validation set. (r= −0.5576, P < 0.01). (**B**) RT-PCR and Q-PCR analysis the effect of miR-122 mimics and SiRNA mediated IGF-1R down-regulation on the expression of MALAT1 in BGC823 gastric cancer cells, β-actin was used as an internal control. (**C**) RT-PCR and Q-PCR analysis the effect of miR-122 inhibitor on the expression of MALAT1 in SGC7901 gastric cancer cells, β-actin was used as an internal control. (**D**), (**E**) RT-PCR, Q-PCR and WB analysis of IGF-1R expression following treatment of BGC823 and CTC141 cells with miR-122 mimics, IGF-1R siRNAs or miR-NC. (**F**), (**G**) RT-PCR, Q-PCR and WB analysis of IGF-1R expression following treatment of SGC7901 and GES cells with miR-122 inhibitor or miR-NC. Data represent the mean ± S.D. from three independent experiments. ***P< 0.001.

The above-mentioned data indicated that MALAT1 was only up-regulated in the GC/DM tissues and plasma. It is still unclear how MALAT1 is regulated in GC cells? We previously reported that miR-122 was significantly down-regulated in GC/DM plasma.[24]. As shown in Figure 5A, the plasma levels of MALAT1 was negatively correlated with that of miR-122 in the validation set (r= −0.5576, *P* < 0.01). We then investigated whether miR-122 could inhibit the expression of MALAT1 in gastric cancer cells. Indeed, enhanced miR-122 expression with miR-122 mimics could inhibit the expression of MALAT1 in BGC823, while miR-122 inhibitor could enhance the expression of MALAT1 in SGC7901 (Figure 5B, 5C). Our unpublished data have indicated that the expression of miR-122 was negatively correlated with that of MALAT1 in gastric cells, while IGF-1R, a target of miR-122, was positively correlated with the MALAT1 level (Unpublished data). As shown in Figure 5D, 5E, 5F, 5G, forced miR-122 expression could inhibit the expression of IGF-1R at mRNA and protein levels both in BGC823 and CTC141, while down-regulation of miR-122 with miR-122 inhibitor could enhance the expression of IGF-1R in SGC7901 and GES. Knockdown of IGF-1R could significantly inhibit the expression of MALAT1 in SGC7901 (Figure 5B). And our unpublished data also indicated that miR-122, as well as IGF-1R knockdown could significantly inhibit cell proliferation, migration and invasion in gastric cancer cells. These preliminary data suggested that the miR-122-IGF-1R axis could regulate the expression of MALAT1 in GC cells. The exact molecular mechanism behind was under further investigation in our lab.

## DISCUSSION

Recent studies have demonstrated that lncRNAs were involved in oncogenesis [5, 6]. The lncRNA MALAT1 is over-expressed in a variety of human tumors. Over-expression of MALAT1 promotes tumor progression [10-17]. In the current study, we demonstrate that circulating lncRNA MALAT1 could be detectable in plasma, and the circulating MALAT1 level was dramatically up-regulated in patients with GC/DM than that of GC/NDM and the healthy controls. Specially, MALAT1 expression was found to be significantly higher at later stages of tumor development and in tumors that had undergone extensive metastasis. There is no difference between the GC/NDM and the healthy controls. Notably, it has been reported that there is no difference in the levels of plasma MALAT1 between gastric cancer patients and healthy controls, and it is unclear whether there is any difference in plasma MALAT1 levels between healthy controls and patients with late-stage gastric cancer in this study [9]. The reason behind may be that the component of the patients were so different in the two studies, and they didn’t take the metastasis state into consideration.

MALAT1 could promote tumor progression through multiple mechanisms in various types of cancer [10-17]. Several recent reports have demonstrated that MALAT1 could function as a promoter of GC cell proliferation and metastasis [25, 26]. We also investigated several potential target proteins of MALAT1, which are involved in cell proliferation, apoptosis, motility and invasion. Knock-down MALAT1 could inhibit the expression of N-cadherin, CyclinD1 and Bcl-xl, which might explain why knock-down MALAT1 could significantly inhibit cell proliferation and invasion in gastric cancer cells (Supple. Figure 3).

Notably, MALAT1 was only up-regulated in GC/DM. The mechanisms underlying the aberrant expression of MALAT1 in gastric cancer remain elusive. The plasma miR-122 was down-regulated in distant metastasis gastric cancer [24]. The presented data indicated that plasma level of MALAT1 was negatively correlated with miR-122. Our unpublished data indicated that the expression IGF-1R, a target of miR-122 was positively correlated with the expression of MALAT1 in gastric cancer cell lines. So we test whether miR-122 could regulate the MALAT1 level in gastric cancer cells. Indeed, miR-122 could inhibit the expression of MALAT1. Further studies indicated that the process of miR-122 down-regulated MALAT1 expression might involve IGF-1R. Previous study has demonstrated that the activated IGF-1R signaling could enhance the activity of β-catenin [27, 28], while recent study indicated that β-catenin could promote the expression of MALAT1 at transcription levels [29]. So our preliminary data combined with several recent studies suggested that the miR-122-IGF-1R axis might participate in the MALAT1 dysregulation in gastric cancer.

In all, the current study indicated that MALAT1 up-regulation might be a valuable biomarker for the distant metastasis in gastric cancer patients, and miR-122-IGF-1R signaling might be involved in the MALAT1 dysregulation in gastric cancer. These findings provided further insight in the gastric tumorigenesis.

## MATERIALS AND METHODS

### Patients and samples

This study was approved by the Clinical Research Ethics Committee of West China Hospital, and all experiments were performed in accordance with relevant guidelines and regulations. All participants provided informed consent. The plasma samples were collected from 72 GC patients and 36 healthy controls. Plasma samples were obtained from the patients at the Department of Abdominal Cancer, West China Hospital of Sichuan University, during the period March 2012 to April 2014. The 36 GC/DM cases included GCs with liver, lung, or bone metastasis, supported by imaging and pathological evidence. Patients were proven the histological diagnosis of GC at initial diagnosis, and none of them had received therapeutic procedures, for instance chemotherapy or radiotherapy. We classified the tumor stage in accordance with the tumor-node-metastasis (TNM) staging system of the Union for International Cancer Control (UICC). The control plasma samples were obtained from individual undergoing a routine physical examination, who showed no evidence of disease. Controls (HCs) were matched to patients by age and gender. The gastric tumor tissues and the adjacent normal tissues (located > 5 cm away from the tumor) were obtained from another 39 GC patients undergoing surgery and snap-frozen in liquid nitrogen for further investigation, at the Department of Abdominal Cancer, West China Hospital of Sichuan University, during the period January 2016 to April 2016. The 25 pairs gastric cancer tissue mainly supported by pathological evidence. Patients were proven the histological diagnosis of GC at initial diagnosis, and none of them had received therapeutic procedures, for instance chemotherapy or radiotherapy. We classified the tumor stage in accordance with the tumor-node-metastasis (TNM) staging system of the Union for International Cancer Control (UICC). The detailed clinical characteristics of study participants are presented in Table S1 and Table S2.

### Plasma preparation and RNA isolation

The total RNA was isolated from the cell lines using TRIzol reagent (Invitrogen, USA). Total RNA was isolated from the tissue samples using NucleoZOL reagent (MN, Germany). Total plasma RNA was extracted by TRIzol LS (Invitrogen, USA), the plasma sample processing and total RNA isolation have been described in our previous work [24].

### qRT-PCR

Reverse transcription reactions were performed using random primers and M-MLV reverse transcriptase kit (Takara, Japan). Real-Tim PCR was performed using a standard SYBR Green PCR kit (Bio-Rad, USA) according the respective manufacturers’ instructions. The primers for MALAT1 were: forward 5′-CTTCCCTAGGGGATTTCAGG-3′ and reverse 5′-GCCCACAGGAACAAGTCCTA-3′, β-actin was used as a loading control in plasma RNA, the primers used for specific β-actin PCR reactions were: forward 5′-TTGTTACAGGAAGTCCCTTGCC-3′, reverse 5′-ATGCTATCACCTCCCCTGTGTG-3′. The primers used for specific miR-122 was from Ribobio (Bulge-LoopTM microRNA qPCR Primer Set) (Ribobio, Guangzhou, China). The primers used for specific IGF-1R PCR reactions were: forward 5′-TGGAGTGCTGTATGCCTCTG-3′, reverse 5′-CCCTTGGCAACTCCTTCATA-3′. GAPDH was used as a loading control in cell RNA, the primers used for specific GAPDH PCR reactions were: forward 5′-CAAGGCCAACCGCGAGAA-3′ and reverse 5′-CCCTCGTAGATGGGCACAGT-3′. Sample was analyzed triplicate.

### Cell culture

The human gastric mucosal cell line GES-1, and the human GC cell lines SGC7901, MKN45 and BGC823 were maintained in our laboratory. The circulating tumor cell lines CTC141 and CTC105 were a kind gift from the Laboratory of Stem Cell Biology of Sichuan University [24]. These two cell lines are CD44-positive cells with great potential for tumor metastasis, and were derived from blood samples of two patients with gastric adenocarcinoma by magnetic isolation, purification and stable passaging. All six cell lines were maintained in RPMI-1640 medium supplemented with 10% fetal bovine serum (HyClone, USA) in 5% CO2 at 37°C.

### Cell transfection

The miR-122 mimic and inhibitor, siRNAs against MALAT1 and IGF-1R were designed and synthesized by Ribobio (Ribobio, Guangzhou, China). The sequence of two siRNAs against MALAT-1 were si-M1 sense 5′-GAGGUGUAAAGGGAUUUAU-3′, si-M2 sense 5′-CACAGGGAAAGCGAGUGGUUGGU-3′and, si-IGF-1R sense, 5′-GAAGAGGUAUUGAAUGCUAdTdT-3′and si-NC sense 5′-CCUACGCCACCAAUUUCGU-3′. And the miR-122and SiRNAs against MALAT1, IGF-1R were transfected into gastric cancer cells respectively by Lipofectamine 2000 (Invitrogen, USA).

### Cell proliferation assay and cell cycle analysis

Gastric cancer cells were seeded in 96-well plates and after 24 hours cells were transfected with two kinds of siRNA or negative control respectively. Relative cell growth was measured by Cell Counting Kit-8 (Dojingdo, Kumamoto, Japan). Cells were transfected with siRNA against MALAT1 for 48 hours. The cells were then digested by trypsin, collected by centrifugation, washed by PBS and fixed overnight at 4°C by 70% ethanol. Then, the cells were washed with PBS and stained by PI at 4oC for 30min according to Cell Cycle Detection Kit (KeyGEN, Nanjin, China). At last, the cells were analyzed with a flow cytometer (BD FACS Calibur, USA)

### Matrigel invasion assay

Twenty-four hours after transfection, cells were collected and suspended in serum-free medium. 4×10^4^ cells in 0.2 ml serum-free medium were plated in the top chamber with matrigel-coated membrane (24-well insert; pore size: 8mm) (BD, USA), with 10% FBS as a attractant. The cells were incubated for 48 h and the filter was stained with hematoxylin and eosine (HE staining) for visualization, and counted.

### Western-blot

Transfected cells were lysed in RIPA buffer (150 mM NaCl, 1% NP-40, 50 mM Tris-HCl PH 7.4, 1 mM phenylmethylsulfonyl fluoride, 1μg/ml leupeptin, 1 mM deoxycholic acid and 1 mM EDTA) containing a cocktail of protease inhibitors and phosphatase inhibitors (Calbiochem, Darmstadt, Germany). Equal amounts of protein sample (30-50 μg) were separated by 12% SDS-PAGE and transferred to PVDF membrane (Millipore, Bedford, MA, USA) using the Bio-Rad semidry transfer system. The following antibodies were used for Western blotting: IGF-1R, N-cadherin, CyclinD1 (Epitomics, USA); Bcl-xl (CST, USA), GAPDH and β-actin (Biostar, China).

### Statistical analysis

The significance of cell and plasma lncRNA levels was determined by the Mann–Whitney, Wilcoxon, and Kruskal–Wallis tests as appropriate. Plasma MALAT1 receiver operating characteristic (ROC) curves were constructed to discriminate among different groups of patients. The area under the ROC curve(AUC) was used to assess the predictive power. The sensitivity and specificity were calculated according to the standard formulas. All of the p-values were two-sided and *p*<0.05 was considered to be statistically significant. All of the statistical calculations were performed using the GraphPad Prism 5.

## SUPPLEMENTARY MATERIAL FIGURES AND TABLES


