# Supplementary material for: The lncRNA MALAT1 is a novel biomarker for gastric cancer metastasis
Source: Oncotarget. 2016 Jul 29;7(35):56209–18. doi: 10.18632/oncotarget.10941 (PMC5302908; doi:10.18632/oncotarget.10941)
Supplement: Supplementary file 1 [file oncotarget-07-56209-s001.pdf]

The lncRNA MALAT1 is a novel biomarker for gastric cancer metastasis

Supplementary Material

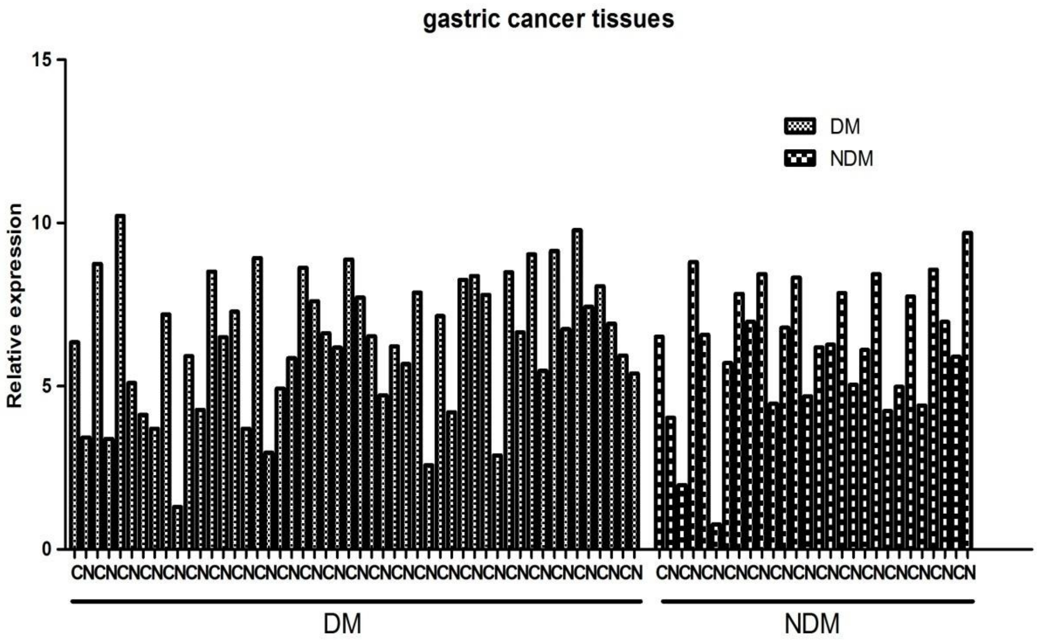

Supplementary Fig. S1. MALAT1 expression was assessed in human gastric cancer tissues and the adjacent normal tissues. Total RNA was extracted from tissues using NucleoZOL, MALAT1 expression was assessed by real-time PCR, and values were normalized to those for β-actin. C represents the cancer tissues and the N represents the adjacent normal tissues. (DM: distant metastasis; NDM: no distant metastasis)

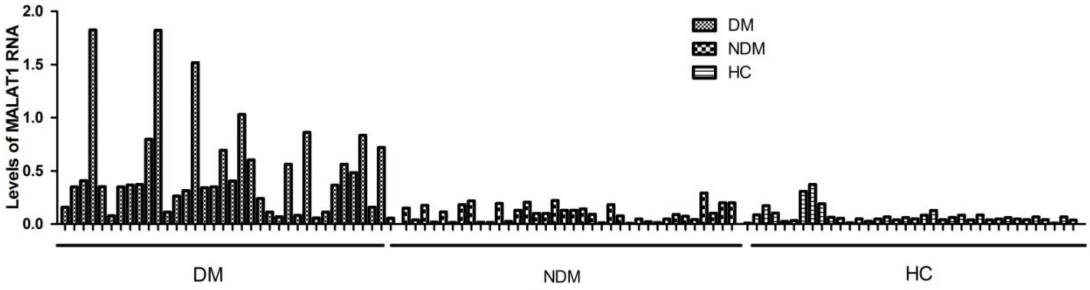

Supplementary Fig. S2. Plasma MALAT1 expression was assessed in human gastric cancer patients and health controls. RNA was extracted from plasma using Trizol-LS. MALAT1 expression was assessed by real-time PCR, and values were normalized to those for β-actin. (DM: distant metastasis; NDM: no distant metastasis; HC: the healthy controls.)

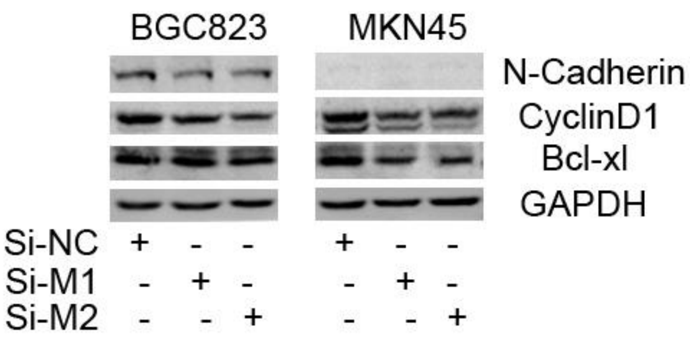

Supplementary Fig. S3. The effect of Si RNA mediated MALAT1 knockdown on the expression of N-cadherin, CyclinD1 and Bcl-xl in gastric cancer cells. GAPDH was used as loading control.

Supplementary Table S1. Clinicopathological features of the GC tissues.

Variables	case number	
	Validation Set(independent samples)	
	**GC/DM(25)**	**GC/NDM(14)**
**Age (years)**		
Median	53	58
Range	30-76	39-77
**Gender**		
Male	16	12
Female	9	2
**TNM stage**		
Ⅲ	0	8
Ⅳ	25	6
**Pathology**		
Well	0	2
Moderate and poor	25	12
**Metastasis location**		
Liver	12	
Lung	8	
Bone	5	
**Lymphnode metastasis**	25	0
**Lauren classification**		
Intestinal type	1	2
Diffuse type	11	0
Mixed type	13	12

Supplementary Table S2. Clinicopathological features of the plasma of GC and the HCs.

Variables	case number		
	Validation Set(independent samples)		
	GC/DM(36)	GC/NDM(36)	HC(36)
**Age (years)**			
Median	56	57	59
Range	48-61	51-66	48-61
**Gender**			
Male	28(77.8)	26(72.2)	28(77.8)
Female	8(22.2)	10(27.8)	8(22.2)
**TNM stage**			
Ⅲ	0(0.0)	13(36.1)	
Ⅳ	36(100.0)	23(63.9)	
**Pathology**			
Well	3(8.3)	4(11.1)	
Moderate and poor	33(91.7)	32(88.9)	
**Tumor location**			
Cardia	14(38.9)	19(52.8)	
Body	7(19.4)	3(8.3)	
Antrum	15(41.7)	14(38.9)	
**Metastasis location**			
Liver	21(58.3)		
Lung	11(30.6)		
Bone	4(11.1)		
**Lauren classification**			
Intestinal type	3(8.3)	4(11.1)	
Diffuse type	33(91.7)	32(88.9)	
**Lymphnode metastasis**			
N1	20(55.6)	18(50.0)	
N2	13(36.1)	14(38.9)	
N3	3(8.3)	4(11.1)	
**Histological type**			
Adenocarcinoma	31(86.1)	33(91.7)	
Other	5(13.9)	3(8.3)	
